# A Combined Approach of Sensor Data Fusion and Multivariate Geostatistics for Delineation of Homogeneous Zones in an Agricultural Field

**DOI:** 10.3390/s17122794

**Published:** 2017-12-03

**Authors:** Annamaria Castrignanò, Gabriele Buttafuoco, Ruggiero Quarto, Carolina Vitti, Giuliano Langella, Fabio Terribile, Accursio Venezia

**Affiliations:** 1 CREA Research Centre for Agriculture and Environment, 70125 Bari, Italy; annamaria.castrignano@crea.gov.it (A.C.); carolina.vitti@crea.gov.it (C.V.); 2 National Research Council of Italy, Institute for Agricultural and Forest Systems in the Mediterranean, 87036 Rende (CS), Italy; 3 Earth and Geoenvironmental Sciences Department, University of Bari Aldo Moro, 700125 Bari, Italy; ruggiero.quarto@uniba.it; 4 Department of Agriculture, University of Naples Federico II, 8055 Portici (NA), Italy; glangella@unina.it (G.L.); fabio.terribile@unina.it (F.T.); 5 CREA Research Center for Vegetable and Ornamental Crops, 84098 Pontecagnano (SA), Italy; accursio.venezia@crea.gov.it

**Keywords:** spatial data, sensor, data fusion, change of support, geostatistics, precision agriculture, management zones

## Abstract

To assess spatial variability at the very fine scale required by Precision Agriculture, different proximal and remote sensors have been used. They provide large amounts and different types of data which need to be combined. An integrated approach, using multivariate geostatistical data-fusion techniques and multi-source geophysical sensor data to determine simple summary scale-dependent indices, is described here. These indices can be used to delineate management zones to be submitted to differential management. Such a data fusion approach with geophysical sensors was applied in a soil of an agronomic field cropped with tomato. The synthetic regionalized factors determined, contributed to split the 3D edaphic environment into two main horizontal structures with different hydraulic properties and to disclose two main horizons in the 0–1.0-m depth with a discontinuity probably occurring between 0.40 m and 0.70 m. Comparing this partition with the soil properties measured with a shallow sampling, it was possible to verify the coherence in the topsoil between the dielectric properties and other properties more directly related to agronomic management. These results confirm the advantages of using proximal sensing as a preliminary step in the application of site-specific management. Combining disparate spatial data (data fusion) is not at all a naive problem and novel and powerful methods need to be developed.

## 1. Introduction

After the post-II World War period, an agriculture model aimed at maximizing yield was elaborated and realized, moving towards intensive agriculture and abandoning extensive agriculture. This change required an increase in both energetic inputs (especially fossil fuel) and chemicals (fertilizers, pesticides) with high toxicity potential. It has been now clearly stated that this way of agricultural management has a negative impact on the environment, reducing soil productivity and causing different types of pollution. This has prompted a search for alternative models with the aim of protecting the environment and its related systems through a reasonable use of natural resources according to sustainable agriculture management. A new approach to sustainable agriculture, called ‘precision farming’ or ‘precision agriculture’, has been developed, aimed at optimizing agriculture productivity while minimizing environmental impact. It is defined as a management strategy that uses information technology to bring data from multiple sources to bear on decisions associated with crop production [1]. This new agricultural model might have a strong economic and environmental impact, since traditional agriculture considers the field as homogenous for physical and chemical characteristics and then applies one treatment. On the contrary, experimental evidence [2,3,4,5] as well as many other references have shown that any agricultural area, even of limited extension, presents spatial variation in soil characteristics; moreover, temporal variability occurs not only throughout the years but also within each crop season.

Precision agriculture is much more than a mere set of even advanced technologies but essentially involves extensive changes in management style and strategy. It aims to match agricultural inputs and practices with the local conditions within a field to help farmers in making better decisions and to achieve their many different goals more efficiently. Therefore, the core of precision agriculture is effectively managing spatial and temporal variability related to all aspects of agricultural production for the purpose of improving crop performance and environmental quality. To obtain such a result, technologies and principles have to be used effectively. Without variability, the concepts of precision agriculture would have little meaning [6] and would never have evolved. Therefore, any precision agriculture system must first address measurement to assess and understand variability. While the technology can facilitate the application of precision agriculture, it is only the knowledge and interpretation of variability that makes it feasible. Therefore, assessing variability is the first critical step and a necessary condition in precision agriculture.

Spatial variability is generally exhibited by a lot of properties and state variables in the natural landscape occurring also over different temporal scales and having different effects on crop growth and yield. Quantifying the variability of these properties adequately can then aid the understanding of the processes causing spatial and temporal variation in crop yield and enlighten the proper management of land resources. 

The availability of efficient and accurate techniques for measuring within-field variations in soil properties at a very fine spatial scale is then required in precision farming [7]. Traditional soil sampling is not viable, because it is costly and labour intensive and needs a large number of soil samples in order to achieve a good representation of soil properties. To overcome the limitations of spatially scarce data, advances in proximal sensing technology and data analysis techniques are now able to provide information on soil, crops and associated environmental properties. Recently, the number of proximal soil sensing techniques (PSS) has increased due to the advantages of non-invasive techniques, which are time- and cost-efficient. In particular, geophysical methods provide a low cost and non-invasive way of gathering large amounts of information on various physical soil properties. At present, a number of soil sensors are available which use a variety of numerous measurement techniques, such as electromagnetic induction (EMI), electrical resistivity (ER), ground-penetrating radar (GPR), gamma sensors, radiometry, fluorimetry, etc.

These sensors can be coupled to a Global Positioning System (GPS) receiver to measure different physical and chemical properties of the soil and plants. However, such measurements are generally affected by more than one agronomic soil characteristic [8] and consequently, obtaining accurate information about one property, by using only one sensing technique, is extremely difficult. Recently, to obtain a more comprehensive representation of the surveyed area and separate more easily the different effects, a new approach for soil and vegetation sensing based on combining several sensing techniques (sensor fusion system), has been developed [9].

Therefore, such an approach requires defining methods to combine information from heterogeneous sources into a single composite picture, which provides more accurate and complete information than that derived from any single source [10]. The task is not easy at all because proximal sensing data are often massive, taken on different spatial and temporal scales, with different spatial resolutions (support sizes) and subject to measurement error biases. Moreover, a statistical approach to data fusion combines the sensor data sets in a manner that is statistically robust because it takes advantage of the differences between the instruments and their complementary features [11,12]. The problem can be treated from different points of view; however, in this work combining multiple sources of information will be handled within a statistical framework. The main advantage of a statistical approach is that explicit probabilistic models are employed to describe the various relationships between the sensors, taking into account the underlying uncertainties and change of support [13]. At present, there exist different statistical methods to jointly analyze heterogeneous data, most of them based on Bayesian [9] and machine-learning methods [14]. Geostatistics, compared with traditional methods, offers the advantage of treating the observations as variables localized in the geometrical and temporal space and to use their degree of autocorrelation to improve estimation precision. Moreover, it provides a measure of the uncertainty of prediction [15].

The objective of this study was to describe an integrated approach by combining multivariate geostatistical data-fusion techniques with multi-source geophysical sensor data, in order to determine simple summary scale-dependent indices that integrate the multi-scale and multi-variate data in a meaningful way. These indices will be used to delineate homogeneous within-field zones (management zones) that could be submitted to differential agricultural management.

## 2. Materials and Methods

### 2.1. Study Site and Soil Sampling

**Figure 1:**
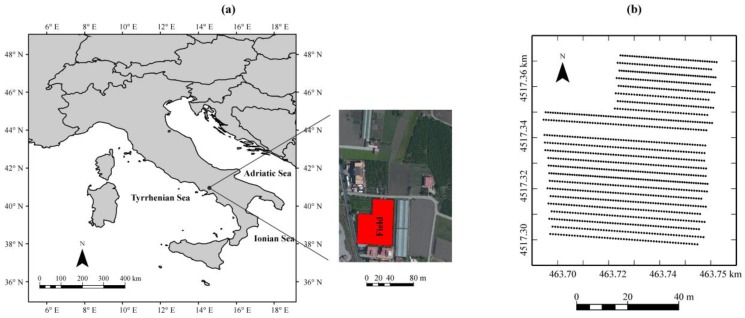
Locations of the experimental field (**a**) and geophysical surveys (**b**). The dotted lines are the 24 transects where the geophysical surveys were carried out.

The study area (Figure 1) is a field located in the Sarno Plain (Striano, Salerno, South-Western Italy) that covers more than 150 km^2^ in the coastal graben of the Campanian Plain. It is bordered by carbonate ridges of the Lattari mountains on the south, the Sarno mountains on the north, the Salerno mountains on the east and the Somma-Vesuvius volcanic complex on the west.

The soils in the alluvial plain landforms at about 14 m a.s.l. develop over alluvial and volcanoclastic deposits. They are typically deep and generally sub-alkaline; the texture ranges from sandy-loam to silty-loam. Considering their intensive agriculture land management, the studied soils have a good supply of organic carbon (min-max in topsoil: 10.15–18.3 g·kg^−1^), available phosphorus (min-max in topsoil: 120–160 mg·kg^−1^) and potassium (min-max in topsoil: 574–879 mg·kg^−1^). The carbonate content is always high and it generally increases with depth. Below 90 cm depth, they can have a partially indurated layer with Fe-Mn concretions, segregation, and mottling induced by either ancient or present day water stagnation.

These soils can be classified as Aquic Calciustepts [16] when they show Fe-Mn hydromorphic features (mottles, segregations, etc.) and Typic Calciustepts [16] when they do not show such hydromorphic features.

The climate is Mediterranean with prevailing winds from north and south and abundant rains in autumn, winter, and spring. Drip irrigation was carried out in summer by pumping water from wells.

The tomato cultivation (cv. San Marzano) was cropped with traditional agronomic treatment following the protected designation of origin (PDO) regulation that echoes the age-old tradition but which is submitted to additional constraints. 

In order to characterize the soil and then give a physical interpretation to the field-partition provided by proximal sensing, 52 soil samples up to a depth of 0.30 m were collected according to grid sampling with mesh of about 10 m. The samples were then submitted to a set of laboratory measurements of the main physical properties. These included texture components by using the pipette method: clay (particle size < 0.002 mm), fine silt (0.002–0.05 mm), coarse silt (0.05–0.02 mm), fine sand (0.02–0.2 mm), coarse sand (0.2–2 mm). In addition, two soil water properties (soil water contents at field capacity and at wilting point) and total carbonate concentration were also determined. 

### 2.2. Data Acquisition and Protocol of Measurements

Geophysical surveys (Figure 1) were carried out on 24 geo-referenced transects approximately 5-m apart along the E–W direction using different sensors connected with a differentially corrected GPS system (DGPS, HiPer 27 Pro, Topcon, Tokyo, Japan) on 16 April 2015. The equipment consisted of:

(1) a monostatic GPR sensor with frequency of 250 MHz (Noggin250, Sensors & Software Inc., Mississauga, ON, Canada);
(2) an electromagnetic induction sensor (EM38DD, Geonics, Ltd., Mississauga, ON, Canada), that provides measurements of either the quad-phase or in-phase component data in both the horizontal and vertical dipole mode;
(3) a multiple frequency electromagnetic profiler GEM300^®^ (Geophysical survey Systems, Inc., Nashua, NH, USA).

Electromagnetic induction (EMI) soil survey is based on the principle that a transmitter coil in contact with the soil surface produces a time-varying primary magnetic field in the subsoil. Any variation in electromagnetic (EM) field induces eddy currents in the soil and generates a secondary magnetic field, which is recorded by a receiver coil in the EM unit. The ratio of the magnitude of the secondary magnetic field to the one of the primary magnetic field allows the calculation of the apparent conductivity near the receiver [17].

The EM38DD EMI sensor, with intercoil spacing of 1 meter and operating frequency of 14.6 kHz, was used to measure bulk electrical conductivity (EC_a_) simultaneously in the horizontal (EC_a H_) and vertical (EC_a V_) polarization modes with different depth response profile: in a homogeneous soil profile, the vertical maximum sensitivity is at a depth of approximately 0.40 m and the signal penetrates to a depth of 1.5 m; whereas the horizontal, maximum sensitivity occurs at the surface and the signal penetrates the topsoil up to 0.75 m. The data were recorded at each second and the spatial resolution was 0.70 m on average along the transects as a function of speed of the tractor which pulled the EMI sensor across the field.

GEM300 Profiler is an EMI sensor, which conceptually works like the previous one. The main difference is the ability to generate at a maximum of 16 pulses in a range of frequencies between 300 and 20,000 Hz. Furthermore, the intercoil distance is 1.67 m and then the maximum impulse response for the vertical dipole mode is about 0.67 m.

The instrument was moved manually along the same 24 georeferenced transects (Figure 1), with the dipole axis of the coils only in a vertical orientation. This choice was determined by the objective of getting deeper information, since the shallow conductivity was already depicted by an EM38DD sensor. The moving speed was about 1 m·s^−1^, collecting in this time six measurements at different frequencies decreasing by a factor of 1.54 (19,975; 12,975; 8425; 5475; 3575, and 2325 Hz). This set of only six operating frequencies was chosen to avoid too long a delay between the sets of measurements and then a too low moving velocity. Furthermore, frequencies less than the smallest one used (2325 Hz) were unnecessary for the agricultural purpose of the survey.

Each operating frequency had a different depth of investigation and therefore explored a different soil volume [18]. The maximum depth of investigation was calculated as the square root of the skin depth choosing a soil conductivity of 50 mS·m^−1^ [19,20]. The skin depth is the effective depth of penetration of electromagnetic energy in a conducting medium when displacement currents can be neglected. It is also the depth at which the amplitude of a plane wave is attenuated to 1/*e* or 37 percent [21].

All the surveys were performed on the same day but separately by sensor type and in soil water conditions close to field capacity after the early spring rainfall events.

Ground Penetrating Radar (GPR) is a noninvasive geophysical tool that is specifically designed to penetrate materials and provide images of subsoil. The radar produces a high-frequency electromagnetic wave, which propagates through the sub-surface materials at the velocity determined by the soil dielectric permittivity. When this propagating wave encounters any change in dielectric properties, it is reflected or scattered back towards the surface where it is recorded [22]. Resistive terrains propagate well the radar waves, while soils with high electrical conductivity (saline or fine textured soils) rapidly attenuate radar energy, which restricts penetration depth and severely limits the effectiveness of GPR. 

The GPR data were collected using the reflection, continuous profiling method, recording one radar trace every 5 centimeters after 16 pulses, along the same transect of the EMI survey. A time window of 100 nanoseconds with 2.5 GHz sample frequency was used.

The processing of GPR data was performed with Gradix Software (Interpex Ltd., Golden, CO, USA) and Radar Software (Sensors & Software Inc., Mississauga, ON, Canada) and included:

(1) a static correction (in this survey only a ‘Set Time Zero’, since the soil surface was flat and the GPR antennas worked on the ground). The best frequency value of a high pass filter (where the amplitude, in the frequency domain, moving towards the low frequencies in the spectrum, begins to increase) was examined and the best number of points searched;
(2) a ‘Dewow filter’ by means of a simple ‘High Pass Filter’ for removing very low frequency components from the data associated with either inductive phenomena or possible instrumentation dynamic range limitations [23];
(3) a ‘Band Pass Filter’ well known for enhancing useful frequency signals. A trapezoidal filter [24] was used for the ‘Band Pass Filter’, which is composed of four cut frequencies, like a trapeze, and was more consistent with the amplitude spectra of our data, after making a frequency analysis.

Before 2D data processing to create time-slices, a procedure called ‘envelope’ was added to improve the attribute analysis for 3D visualizations [20,25,26]. A quadrature filter (Hilbert-Transformation) was used to calculate the instantaneous signal amplitude or envelope of data, which gives an estimation of the reflectivity and is proportional to the square root of the total energy of the signal at a given instant of time [27]. After the application of this procedure, the signal amplitude becomes a relative quantity and gives an overview of the distribution of the underground reflectors (soil layering, in the case of applications for agriculture), their reflectivity or dielectric contrasts, and the intrinsic attenuation of the media or their physical-chemical characteristics (soil structure, mineralogy, water content, salinity).

The mean signal amplitude was calculated within a range of 4 ns (the period at the frequency of 250 Mhz) by increasing time intervals, which overlapped (0–4 ns, 2–6 ns, 4–8 ns and so on) up to 40 ns, where the signal shrinks sharply [20]. A depth conversion was performed by means of the analysis of hyperbolae occurring on the radar sections. A mean value of about 10 cm·ns^−1^ was calculated and then the depth intervals were of 20 cm (0–20 cm, 10–30 cm, 20–40 cm and so on), since the above mentioned time is actually a two way travel time of the radar waves.

### 2.3. Geostatistical Analysis

To jointly analyze the heterogeneous data set, including data from the different sensors mostly not co-located and with different support and level of uncertainty, a complex but well integrated approach of geostatistical procedures was defined including the following steps:

(1) Sample Data Migration

To perform multivariate analysis on different sensor data, the raw data of GEM and GPR were collocated into the less numerous file containing EM38DD measurements by migrating them to the nearest EMI sample point up to a maximum distance of 5 m (approximately the distance between the transects);

(2) Gaussian Anamorphosis Modelling

All variables were normalized through a Gaussian anamorphosis [28,29] to zero mean and unity variance to solve the two-type problems occurring in: (a) multivariate approach when the variables differ widely in size and (b) variogram modelling owing to the presence of outliers in highly skewed data distributions. Gaussian anamorphosis is a mathematical function, which transforms a variable *Y*, with a Gaussian standardized distribution, into a new variable *Z* with any distribution:

(1) Z(x)=φ[Y(x)]

where **x** is the location coordinates vector. As the function φ[Y(x)] needs to be known for any Gaussian value, it is modelled by fitting a polynomial expansion [28]:

(2) $$\varphi \left[Y\left(x\right)\right]=\sum_{i=0}^{n}\psi_{i}\;H_{i}\left[Y\left(x\right)\right]$$

where $H_{i}\left[Y\left(x\right)\right]$ are called Hermite polynomials. In practice, the polynomial expansion is truncated at a generally high order (30–100) and tends to be bijective within the interval defined by the minimum and the maximum of the sample values [29]. Model fitting then consists of calculating the *Ψi* coefficients of the expansion; in order to transform the raw variable into a Gaussian one, the anamorphosis function has to be inverted [29].

All the geostatistical techniques were performed on the transformed data and at the end, the estimates were back-transformed to the raw values of the variables through the anamorphosis functions previously calculated;

(3) Fitting of Linear Model of Coregionalization

The linear model of Coregionalization (LMC), developed by Journel and Huijbregts [30], considers all the *n* studied variables as the result of the same independent physical processes, acting over different spatial scales *u*. The *n* (*n* + 1)/2 simple and cross semivariograms of the variables are modelled by a linear combination of *N_S_* standardized semivariograms of unit sill, $g^{u}\left(h\right)$. Using the matrix notation, the LMC can be written as:

(3) $$\Gamma \left(h\right)=\sum_{u=1}^{N_{S}}B^{u}g^{u}\left(h\right)$$

where $\Gamma \left(h\right)=\left[\gamma_{ij}\left(h\right)\right]$ is a symmetric matrix of order *n* × *n*, whose diagonal and out-of-diagonal elements represent simple and cross variograms, respectively; $B^{u}=\left[b_{ij}^{u}\right]$ is called the coregionalization matrix and is a symmetrical positive semi-definite matrix of the order *n* × *n* with real elements $b_{ij}^{u}$ at a specific spatial scale *u*. The model is properly defined if the functions $g^{u}\left(h\right)$ are authorized variograms models [31];

(4) Block (Co)kriging and Change of Support.

The solution to most problems of change of supports requires spatial prediction. A common inferential problem consists in predicting a random variable *Z* defined over a block *B* from *N* point samples $Z(x_{i})$. In linear geostatistics, the predictor is called block kriging and is given by:

(4) $$Z(B)=\sum_{i=1}^{N}\lambda_{i}Z(x_{i})$$

where optimal weights *λ* are obtained by solving kriging system equations resulting from minimizing prediction mean squared error subject to unbiasedness constraint [28]. The equations are very similar in point- and block kriging; the main difference consists in the calculation of the point-to-block covariance:

(5) $$\bar{C}(B,x_{i})=\mathrm{cov}\left(Z(B),Z(x_{i})\right)=\int_{B}C(v,x_{i})du\prime /\left|B\right|$$

where $\bar{C}$ is point-to-block covariance, |B| is the volume of the block which is called spatial support and *C* is point-to-point covariance.

In practice, $\bar{C}$ is approximated by a summation because *B* is discretized into points (*u*’) to compute the integrals. Given the observations at locations with point support, block kriging can be used to predict the average value over a bigger support, accounting for size but also for shape and orientation of the blocks (support). Therefore, block kriging can be considered as a solution to the problem of change of support. 

Cokriging is a multivariate spatial predictor, which was developed to improve the prediction of an ‘undersampled’ variable by determining the spatial correlation with other variables, which are more easily and extensively measured. The optimal ordinary cokriging predictor is a linear combination of all available data and, similarly to kriging, its equations result from minimizing the prediction mean squared error subject to unbiasedness condition. The main difference is that autocovariance and cross-covariance (between two variables) functions need to be estimated. The interested reader can find the full details of cokriging in Wackernagel [29] and Webster and Oliver [32]. The equations are valid regardless of the support of data, however taking into account different supports in the calculation of autocovariance and cross-covariance functions is crucial to obtain valid inference. Once a consistent model for the point covariance functions is estimated, using LMC as described above, block cokriging can be applied by replacing the cross-covariances in point cokriging by their block-averaged counterparts through variogram regularization [33]. The aim of variogram regularization is to transform a variogram model established on points into the corresponding variogram on a given block support. To build a regularized variogram, it needs to have a variogram model fitted on the raw or Gaussian point variable; the model can be also multivariate. The regularized variogram will then be calculated in different directions using a discretization of the blocks into equal cells, where the blocks are replaced by the union of the cell centers. Therefore, regularization consists in calculating a pseudo-experimental variogram on fictious points regularly discretized over the new support. Finally, it will be necessary to fit a new model on the block variogram before using it in the subsequent procedures of kriging or cokriging.

In summary, block cokriging can be used to solve both point-point and point-block problems of change of support although it can be easily extended to solve any type of change of support problems [34].

Finally, factorial block cokriging was applied to analyze the correlation structure between the variables by applying principal component analysis (PCA) at each spatial scale *u*, to obtain independent factors which synthesize the multivariate information, to estimate the values of these specific factors at each characteristic scale using block cokriging, and then to map them.

The regionalized principal component analysis breaks down each co-regionalization matrix $B^{u}$ into eigenvalues and eigenvector matrices [25]:

(6) $$B^{u}=Q^{u}\Lambda^{u}Q^{uT}=\left(Q^{u}\sqrt{\Lambda^{u}}\right){\left(Q^{u}\sqrt{\Lambda^{u}}\right)}^{T}=A^{u}A^{uT}$$

where Q*^u^* is the matrix of eigenvectors, i.e., the regionalized factors $Y_{v}^{u}\left(x\right)$, and Λ*^u^* is the diagonal matrix of eigenvalues for each spatial scale *u*; $A^{u}=Q^{u}\sqrt{\Lambda^{u}}$ is the matrix of order *n* × *n* of the transformation coefficients $a_{iv}^{u}$, which correspond to the covariances between the original variables $Z_{i}\left(x\right)$ and the regionalized factors $Y_{v}^{u}\left(x\right)$.

The approach breaks down the set of original second-order random variables $\left\{Z_{i}\left(x\right);i=1,\ldots ,n\right\}$ into *N_s_* sets of reciprocally orthogonal factors $\left\{Y_{v}^{u}\left(x\right);v=1,\ldots ,n;u=1,\ldots ,N_{s}\right\}$ with transformation coefficients $a_{iv}^{u}$ for each spatial scale *u*. Combining the spatial with the multivariate decomposition each variable *Z_i_* can be written as:

(7) $$Z_{i}\left(x\right)=\sum_{u=1}^{N_{S}}\sum_{v=1}^{n}a_{iv}^{u}Y_{v}^{u}\left(x\right)$$

Mapping the regionalized factors $Y_{v}^{u}\left(x\right)$ provides an illustration of the joint behavior of the variables and their spatial relationships at the different spatial scales. The estimation of the factors is performed by solving a modified cokriging system, as described by Wackernagel [29] and Castrignanò et al. [31].

All geostatistical analyses were performed using the software Isatis 2017.1 (Geovariances, Avon Cedex, France) [35].

## 3. Results and Discussion

The summary statistics of the selected twenty-eight geophysical variables from the three different sensors, which will be used for field delineation, are reported in Table 1.

From the inspection of Table 1, it is evident that all variables generally show large variability; in more detail GEM variables look quite similar, though with a tendency to decrease on average at lower frequencies, due to the larger volume of soil (support) surveyed by the electromagnetic (EM) signal. However, the EM wave at 3575 Hz shows a drastic reduction in the mean value of bulk EC, which can be explained in terms of soil properties. The same trend appears in the two variables from the EM38DD sensor: a large decrease of mean bulk EC in depth (vertical polarization) jointly with an increase of variation.

As regards the GPR data at the different depths, a tendency of reduction of the signal amplitude occurs in depth, as is expected owing to the attenuation, especially at depths deeper than 0.60–0.70 m; beneath 1.0-m depth, the signal was critically attenuated. 

The values of skewness and kurtosis show that the data distributions are mostly positively skewed, with the exception of the shallower GPR data up to 0.30 m-depth (negatively skewed), and sharply peaked (high kurtosis), which is evidence of great uniformity but with the presence of a few large outliers. Before performing Gaussian anamorphosis, most of the outliers were filtered, especially the ones located along the lower western border, owing to the presence of a metallic gate in the field. The goodness of fitting of Gaussian modelling was tested by cross-validation producing mean errors quite close to 0 and standard deviations of error less than 10% of the standard deviation of the corresponding raw variable.

**Figure 2:**
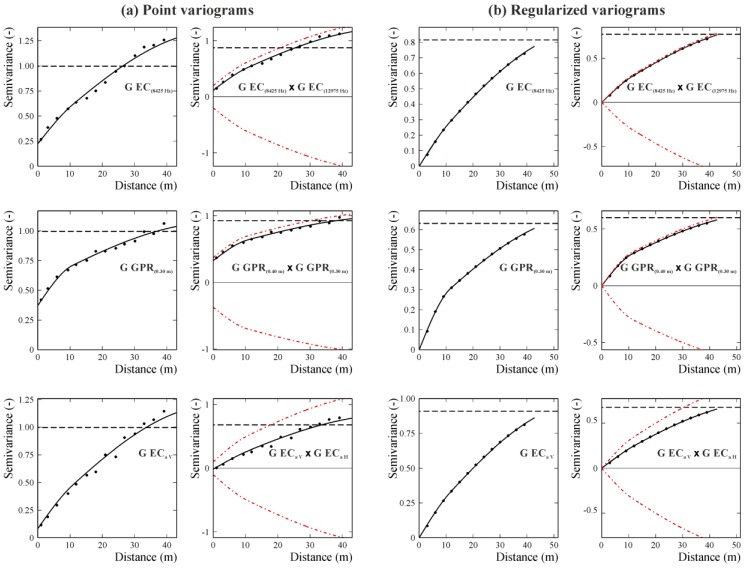
Examples of point (**a**) and reguralized (**b**) auto- and cross-variograms of the Gaussian variables of EC_a(- Hz)_ (apparent electrical conductivities measured by the GEM Profiler), EC_a H_ and EC_a V_ (apparent electrical conductivities measured by the EM38DD in horizontal and vertical polarization) and GPR signal amplitude at different depth slices. The experimental values are the plotted points and the solid lines are of the model of coregionalization. The dash-dotted lines are the hulls of perfect correlation and the dashed lines are the experimental variances.

As no heteroscedasticity effect of variance with direction was manifest in the experimental point variograms, an isotropic LMC model was fitted to the direct and cross-variograms of the twenty-eight Gaussian transformed variables, consisting of the three following basic spatial structures: a nugget effect, a spherical model with a range of 10.74 m and a spherical model with a range of 55 m. Then, the data were interpolated with block cokriging on a regular grid with a mesh of 1 m, after regularization of the previous point LMC using block size equal to the grid mesh. Regularization did not change the type of the mathematical models but modified the number of the structures by eliminating the nugget, reduced the sills, and increased the ranges slightly. In more details, the regularized LMC included two basic structures: a spherical model with range =11.66 m and a spherical model with range =60.55 m. The associated variances to the two spatial structures were quite comparable though a light prevalence for the structure at longer scale, as it results from the cumulative values of their corresponding eigenvalues. The effect of regularization was showed in Figure 2, where, as an example, some of the point variograms compared with the corresponding regularized variograms are reported.

The goodness of fitting was satisfactory as the mean errors were close to zero and variances of the standardized errors close to 1 (data not shown).

**Figure 3:**
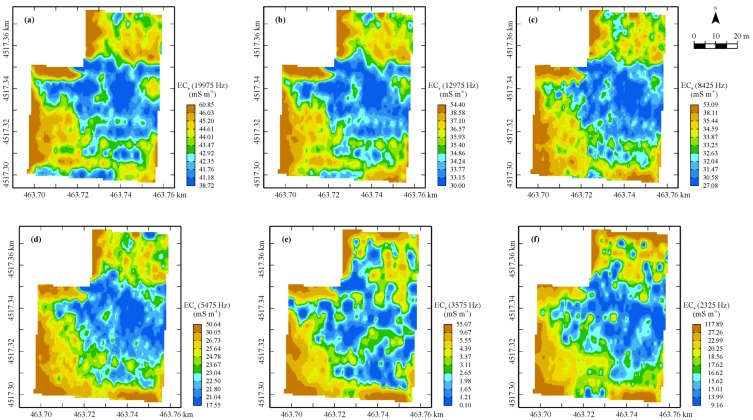
Maps of estimates of EC_a_ (mS·m^−1^) at the selected frequencies (**a–f**). Color scale uses iso-frequency classes.

**Figure 4:**
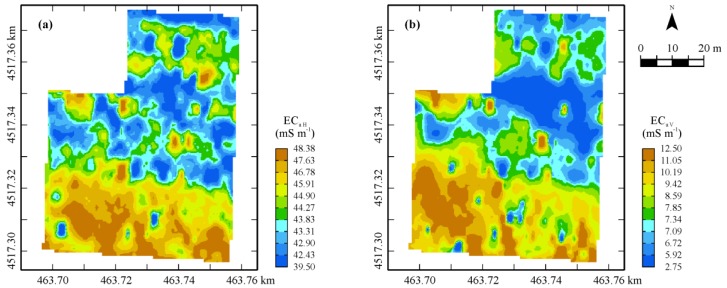
Maps of estimates of EC_a_ (mS·m^−1^) in the (**a**) horizontal and (**b**) vertical orientations. Color scale uses iso-frequency classes.

**Figure 5:**
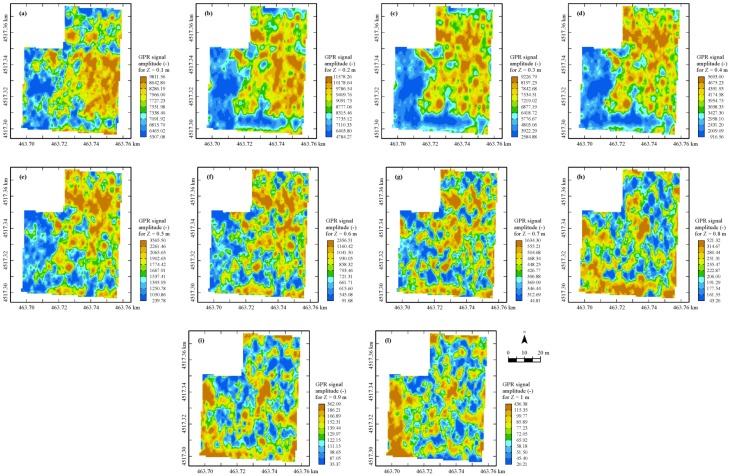
Maps of horizontal sections of estimated GPR signal amplitude envelope. Only a selection up to 1 m of the horizontal sections (**a–l**) are reported. Color scale uses iso-frequency classes.

The maps of the geophysical variables are shown in Figure 3, Figure 4 and Figure 5: a great similarity among the six bulk EC_a_ maps (Figure 3) from the GEM sensor is evident, with a wide central area (blue) characterized by the lowest values (the least conductive zone), and three less extensive but more conductive zones (brown) north, south, and along the western border of the field (Figure 3a). As regards the map corresponding to the frequency of 3575 Hz, it is evident an expansion of the central lower-value area (blue) is at the expense of the other three (brown), with a reduction of the overall minimum and mean values compared with the corresponding values of the other GEM maps but not of the maximum values. What is observed above, is indicative of some sort of discontinuity along the profile up to 2.0 m-depth, though it is not possible from the only cumulative EMI measurements to localize where it occurs. This finding could be associated with the evidence that in the area two soils (Aquic Calciustepts and Typic Calciustepts) coexist, either having or not having in the subsoil Fe-Mn concretion/segregations and mottles.

The EC_a_ maps in both orientation modes look similar enough to the previous ones, although the area characterized by lower values (blue) is much larger and extends also towards the northern part of the field (Figure 4). 

The zone with the highest values (brown) in the map in horizontal mode, which are comparable with the ones of EC_a_ maps from the GEM sensor, is restricted only to a strip in the southern part of the field. It is a little surprising to see the large decrease in bulk EC_a_ values for the mode in vertical polarization, which is a further indication of the lack of soil continuity in depth in terms of moisture and/or granulometry and/or structure. Again, this type of sensor, which provides an integrated signal along the surveyed soil profile, is not capable of localizing the discontinuity and displaying the vertical stratification. This was the reason why it was decided to integrate the previous EMI sensors with a different type of geophysical sensor, a GPR, whose outcomes can be localized in a 3D space.

In Figure 5, the horizontal sections of block cokriging estimates of GPR signal amplitude at each 0.10-m depth-increment are shown. The maps appear sufficiently similar and in some way reversed compared to EC_a_ maps, at least up to about 0.60-m depth.

This behavior is quite predictable, since the more conductive areas cause greater attenuation of the radar signal. The spatial pattern changes drastically at depths deeper than 0.60 m, becoming quite erratic without clear structures of spatial dependence, which is indicative of some discontinuity along the profile occurring at this depth. At depths deeper than 1 m the GPR looked strongly attenuated, therefore the corresponding maps were not displayed and any further discussion was restricted to the shallow 1-m depth of soil. The advantage of including GPR in the multi-sensor set then appears evident, compared to an EMI sensor, because it allows one to detect significant structural changes along the soil profile, which can affect the conditions of root development and the nutrient and water uptake of the tomato crops grown in the field.

In order to obtain a delineation of the field into homogenous areas, among the block factorial cokriging results, the shorter-range structure was ignored because it is related to variability occurring at a scale (about 11 m) deemed to be too small for actual agronomic management. Therefore, only the first two regionalized factors of the spherical model at longer range (60 m), corresponding to eigenvalues greater than 1, were retained and mapped.

The first factor is mainly affected positively by the outcomes of the eight EMI sensors (both GEM and EM38 DD sensors) and negatively by the more superficial GPR outcomes up to the depth of 0.30 m, explaining about 64% of the variance at the 60-m scale (Table 2). It could then be interpreted as a synthetic indicator of the conductive properties of the shallow 0.30-m depth. On the second factor, the GPR outcomes between 0.40 and 0.60 weigh mainly and positively and, to a less extent, the other GPR outcomes at deeper depths, explaining only about 18% of the variance at the same spatial scale (60 m). These results show clearly that some structural change is occurring along the vertical profile within the transition 0.40–0.60-m horizon of the soil.

**Figure 6:**
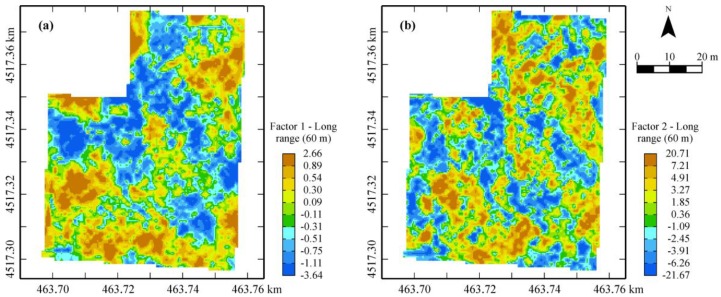
Maps of the first (**a**) and second (**b**) regionalized factors from sensor data fusion.

Figure 6 shows the maps of the two previous factors that realize the decomposition of the 3D spatial variance of the soil into two orthogonal components: the first related to the shallow soil (0–0.30 m depth) horizon; the second to depths deeper than 0.30 m at least up to 1.0 m, owing to the strong attenuation of the GPR signal amplitude below this depth.

The map of the first factor displays a wide central area characterized by lower values (blue) of the EMI sensor outcomes but less attenuation of the GPR signal amplitude, probably ascribable to better structural properties of the shallow soil (0–0.30 m-depth). In the map of the second factor, the main structures of the spatial dependence are less clearly defined; however, a tendency can be observed towards lower values of GPR outcomes in the central area (blue) and towards higher values at the northern and south-western parts of the field (brown). Remembering the interpretation of the factors described above (Table 2), the two factors can be considered as synthetic indicators of the dielectric properties of the two different horizons of soil (above and below 0.30-m depth), confirming the occurrence of some sort of discontinuity along the 1.0-m soil profile.

Summing up, the maps of the two factors graphically realize the fusion of the three sensors outputs, providing a partition of the field into zones with different dielectric properties of the soil at different depths. However, in order to interpret the variations shown in the previous maps in terms of actual soil characteristics, which could have an impact on agronomic management, a direct soil sampling of the shallow 0–0.30-m depth was necessary.

The measured topsoil properties of the soil under study are reported in Table 3. Soil is sandy-loam with a coarse sand and silt fractions on average respectively greater than 55% and 32%; this balanced texture provides the soil with good water draining and retaining properties, as reported by the mean values of the wilting point (WP) and field capacity (FC). Moreover, the soil has a good carbonate content, which also contributes to improving the soil structure [36]. From the correlation matrix (Table 4), it is shown that the total carbonate is significantly and positively correlated with most of the other variables with the exception of coarse sand which, conversely, is negatively correlated with all the other variables, showing a sort of antithetic behavior between the two soil properties (total carbonate and coarse sand). The water retaining properties of soil at low pressure (wilting point) are positively affected by both total carbonates and total silt content (which are positively correlated between them); whereas at high pressure (field capacity) the soil behavior is positively affected only by the fine component of silt. A clear interpretation of the contribution of silt to the hydrologic properties of our soils is difficult to evaluate. This is because fine and medium silt fractions are indeed the main grain sizes of pyroclastic material, but our soils have also a clear alluvial parent material contribution (with other coarser silt fractions) which makes the particle size distribution of the parent material quite varied.

Moreover, fine sand and coarse silt seem to show a very similar behavior, representing a transition phase for the granulometric and structural properties of soil (from coarse sandy to finer textured soils). Summing up, the observed combination of total carbonate content and texture of the topsoil seems to favor the good properties of water retention and drainage observed for these soils.

The occurrence of the previous significant correlations between the soil properties justifies the multivariate approach that was performed. As all variables showed significant departure from Gaussian data distribution, they were previously Gaussian transformed and standardized to mean 0 and variance 1.

An isotropic LMC was fitted to all experimental variograms including the following spatial structures: nugget effect, spherical model with range =16.3 m and spherical model with range =33.8 m, which realize a decomposition of the total variance in three components: uncorrelated error (about 35%), micro-scale variance (about 18%), longer-scale variance (about 46%). Therefore, more than 50% of the total variance is ascribable to variation occurring to a scale (<16.3 m) that is hardly manageable by tillage or other agricultural procedures. Only about 46% of the total variance, related to spatial scale of 33.8 m, can be attributed to more structural properties of soil.

Point factorial cokriging (as all soil variables have the same support) was then applied exclusively to the longer-range structure of the previous LMC and the first factor was retained because it was associated with an eigenvalue greater than 1, explaining more than 62% of the variance at this scale. From the description of the structure of this factor (Table 5), it follows that the variables weighing more on it are: positively, total carbonates and wilting point; negatively, coarse sand. 

**Figure 7:**
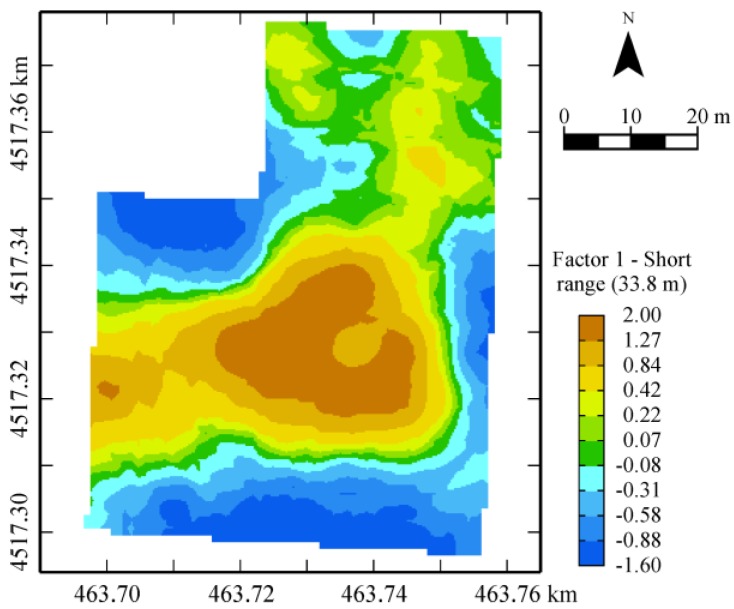
Map of the 33.8-scale factor of soil properties.

**Figure 8:**
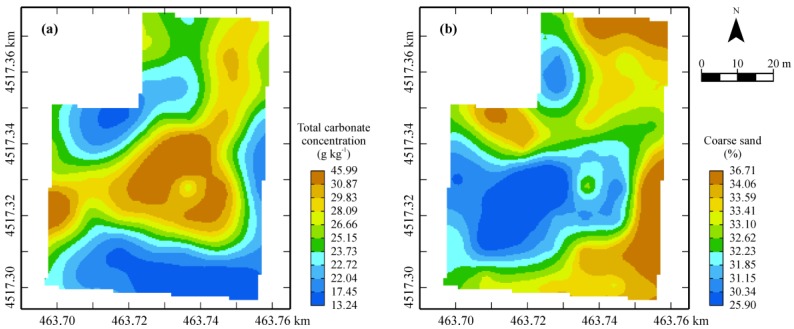
Cokriged maps of total carbonate (**a**) and coarse sand (**b**).

The map of the first factor (Figure 7) shows a wide central area (brown) characterized by the highest carbonate contents and the minimum contents of coarse sand (Figure 8). The high content of carbonates (Figure 8) gives soil good structural properties, improving also water drainage and retention.

Comparing Figure 7 of the topsoil factor with Figure 6a of the first factor of the geophysical variables, which is mainly affected positively by EMI sensor outcomes and negatively by GPR amplitude in the depth 0–0.30 m (Table 2), it is possible to verify a perfect coherence between them. In fact, the high contents of carbonates increase soil resistivity (low bulk EC) and reduce attenuation of EM signal (high GPR signal amplitude outcomes).

As shown before, the dielectric properties of soil are inverted in depth (>0.30 m) (Figure 5); the correspondence with actual soil properties could be verified only with soil profiling, because soil sampling was carried out only in the shallow 0–0.30 m depth more interest for tomato rooting.

**Figure 9:**
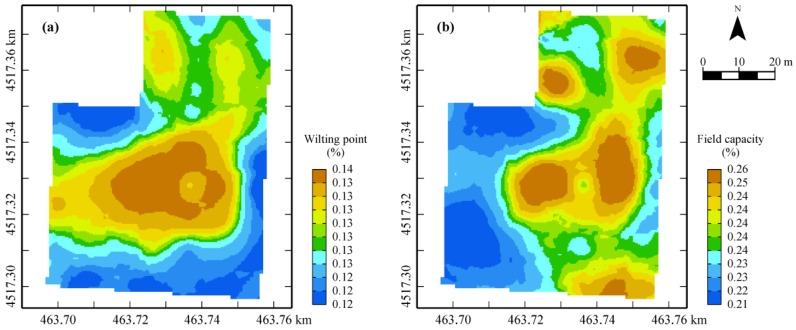
Cokriged maps of wilting point (**a**) and field capacity (**b**).

The occurrence of this wide area in the central-northern part of shallow soil, creating better water conditions for plants, as verified by higher values (brown) of wilting point and field capacity (Figure 9), can clearly have an impact on crop growth and water requirements, making site-specific management necessary.

The different response of the crop to the varied edaphic conditions could be tested through a remote (satellite, aero or UAV) image of the field, which was not carried out. However, it was proven that what was sensed by a set of different geophysical sensors can be attributed to real physical properties of the soil capable to impact agricultural management.

## 4. Conclusions

A data fusion approach based on multivariate geostatistics, for combining spatial data of different types from geophysical sensors, was described and applied in a soil of an agronomic field cropped with tomato. The calculated synthetic regionalized factors contributed in splitting the 3D edaphic environment into two main horizontal structures with different hydraulic properties and to disclose two main horizons in the 0–1.0-m depth with a discontinuity probably occurring between 0.40 m and 0.70 m. Comparing this partition with the soil properties measured with shallow sampling, it was possible to verify the coherence in the topsoil between the dielectric properties and other properties more directly related to agronomic management. These results confirm the advantages of using proximal sensing as a preliminary step in the application of site-specific management, even if the interpretation of field delineation, obtained by soil sensing in terms of effective management zones, can be done only through direct sampling and field scouting. 

Finally, it is worth underlining that combining disparate spatial data (data fusion) is not at all a naive problem and novel and powerful methods should be developed. Although different solutions have already been proposed [37], very few techniques treat the fundamental problem of change of support, associated with the joint analysis of different sensors, explicitly. The geostatistical way of facing the problem is to estimate cross-covariance functions of a multivariate data set over different sample size (block), as was performed in this work with the regularization of LMC used in block cokriging and block factorial cokriging. In some cases, more complex non-linear approaches can still be required, but a measure of prediction uncertainty, as geostatistics effectively does, is needed. What was stated beforehand, points out the necessity of promoting research to solve the problem of change-of-support in sensor data fusion, common to many other disciplines in our time, characterized by an abundance of multi-source information.

## Figures and Tables

**Table 1:** Basic statistics of the selected geophysical variables used in the study. EC_a(- Hz)_ are the apparent electrical conductivities (mS·m^−1^) measured by the GEM Profiler at the different operating frequencies; EC_a H_ and EC_a V_ are the apparent electrical conductivities (mS·m^−1^) measured by the EM38DD in horizontal (H) and vertical (V) polarization; GPR_(- m)_ are the GPR signal amplitude (-) at the different depth slices.

Variable	Minimum	Maximum	Mean	Median	Stand. Dev.	Skewness	Kurtosis
EC_a(19,975 Hz)_	34.92	122.49	43.94	43.43	4.19	9.28	162.41
EC_a(__12,975 Hz)_	29.60	106.76	35.94	35.37	4.39	7.19	97.88
EC_a(__8425 Hz)_	26.02	103.407	34.22	33.26	5.69	6.33	64.40
EC_a(5475 Hz)_	17.49	149.06	25.27	23.80	7.84	8.75	114.65
EC_a(3575 Hz)_	0.10	200.29	5.38	3.32	11.75	12.14	185.23
EC_a(2325 Hz)_	6.03	294.03	21.34	18.11	16.28	9.07	124.96
EC_a H_	39.50	66.88	44.86	44.38	2.72	2.82	17.76
EC_a V_	2.75	73.13	8.52	8.00	3.84	8.50	124.74
GPR_(0.10 m)_	1260.00	10,387.00	7315.03	7481.50	1332.98	−1.08	5.42
GPR_(0.20 m)_	1840.00	12,333.00	8191.51	8492.00	1953.14	−0.52	2.88
GPR_(0.30 m)_	1115.00	10,234.00	6192.77	6515.00	1957.09	−0.30	2.12
GPR_(0.40 m)_	634.00	7042.00	3420.70	3521.50	1248.71	0.01	2.29
GPR_(0.50 m)_	239.00	5628.00	1658.09	1619.50	667.15	0.58	4.25
GPR_(0.60 m)_	91.00	3792.00	845.47	775.00	390.55	1.28	7.60
GPR_(0.70 m)_	30.00	2085.00	439.04	427.00	191.97	1.96	14.63
GPR_(0.80 m)_	24.00	1258.00	241.15	225.00	113.79	2.13	15.30
GPR_(0.90 m)_	18.00	751.00	143.91	135.00	72.70	1.76	11.11
GPR_(1.00 m)_	14.00	434.00	83.99	75.00	47.31	2.03	11.15
GPR_(1.10 m)_	11.00	299.00	55.55	47.00	33.42	2.39	12.46
GPR_(1.20 m)_	10.00	229.00	49.96	44.00	27.35	1.85	8.77
GPR_(1.30 m)_	5.00	149.00	43.07	38.00	22.93	1.30	5.30
GPR_(1.40 m)_	2.00	136.00	31.65	28.00	18.84	1.65	7.26
GPR_(1.50 m)_	1.00	98.00	23.37	19.00	14.08	1.53	6.24
GPR_(1.60 m)_	2.00	71.00	19.08	17.00	10.34	1.14	4.44
GPR_(1.70 m)_	1.00	69.00	15.79	14.00	8.24	1.14	5.47
GPR_(1.80 m)_	0.00	50.00	12.12	11.00	6.17	1.10	5.35
GPR_(1.90 m)_	0.00	30.00	8.90	8.00	4.44	0.90	4.36
GPR_(2.00 m)_	0.00	27.00	6.48	6.00	3.33	0.99	5.38

**Table 2:** Structural composition of the first two regionalized factors of the spherical model at longer range (60 m) with eigenvalue and explained variance (%).

Variable	Factor 1	Factor 2	Variable	Factor 1	Factor 2
EC_a(19__,__975 Hz)_	0.3012	0.1436	GPR_(0.70 m)_	−0.0457	0.2016
EC_a(12,975 Hz)_	0.3282	0.1280	GPR_(0.80 m)_	0.0534	0.1673
EC_a(8425 Hz)_	0.3432	0.1052	GPR_(0.90 m)_	0.0264	0.2113
EC_a(5475 Hz)_	0.3471	0.0980	GPR_(1.00 m)_	0.0774	0.1595
EC_a(3575 Hz)_	0.2772	0.1348	GPR_(1.10 m)_	0.1044	0.1699
EC_a(2325 Hz)_	0.2987	0.1317	GPR_(1.20 m)_	0.0908	0.1614
EC_a H_	0.2464	−0.0880	GPR_(1.30 m)_	0.0639	0.1445
EC_a V_	0.2991	−0.1266	GPR_(1.40 m)_	0.0423	0.1699
GPR_(0.10 m)_	−0.2161	−0.0707	GPR_(1.50 m)_	0.0325	0.1640
GPR_(0.20 m)_	−0.2396	0.1463	GPR_(1.60 m)_	−0.0071	0.1602
GPR_(0.30 m)_	−0.2178	0.3279	GPR_(1.70 m)_	−0.0447	0.1621
GPR_(0.40 m)_	−0.1797	0.4011	GPR_(1.80 m)_	−0.0579	0.1651
GPR_(0.50 m)_	−0.1120	0.3634	GPR_(1.90 m)_	−0.0137	0.1502
GPR_(0.60 m)_	−0.0806	0.2802	GPR_(2.00 m)_	0.0301	0.0747
Eigenvalue				6.3576	1.7734
Variance Percent				63.74	17.78

**Table 3:** Basic statistics of soil properties.

Variable	Minimum	Maximum	Mean	Median	Stand. Dev.	Skewness	Kurtosis
Total carbonates (g·kg^−1^)	13.21	46.22	25.37	24.39	5.93	0.79	4.87
Clay (%)	9.09	17.46	12.38	12.38	1.48	0.50	4.71
Coarse sand (%)	24.96	37.95	32.48	32.48	2.34	−0.58	4.67
Coarse silt (%)	12.39	16.92	13.77	13.62	0.84	0.98	4.96
Fine silt (%)	14.35	28.87	18.00	18.65	2.69	1.47	6.02
Fine sand (%)	19.23	27.25	22.71	22.71	1.55	0.31	3.10
Field capacity (%)	0.14	0.35	0.24	0.24	0.04	0.26	4.18
Wilting point (%)	0.10	0.19	0.13	0.13	0.01	1.31	10.22

**Table 4:** Correlation matrix of soil variables.

Variable	Total Carbonates	Clay	Coarse Sand	Coarse Silt	Fine Silt	Fine Sand	Field Capacity	Wilting Point
Total carbonates	1.00							
Clay	0.05	1.00						
Coarse sand	−0.32	−0.26	1.00					
Coarse silt	0.22	0.21	−0.21	1.00				
Fine silt	0.23	−0.40	−0.46	−0.47	1.00			
Fine sand	−0.08	0.01	−0.35	0.39	−0.40	1.00		
Field capacity	0.08	−0.02	−0.13	−0.20	0.36	−0.31	1.00	
Wilting point	0.38	0.04	−0.35	0.14	0.35	−0.19	0.13	1.00

**Table 5:** Structural composition of the first regionalized factor of the spherical model at longer range (33.80 m) with eigenvalue and explained variance (%).

Variable	Factor 1
G Field capacity	0.0939
G Wilting point	0.3758
G Coarse sand	−0.4416
G Coarse silt	0.2533
G Fine sand	−0.0031
G Silt	0.2766
G Total carbonate	0.7171
Eigenvalue	2.0347
Variance Percent	62.24
